# Supplementary material for: Tracking Antimicrobial Resistant E. coli from Pigs on Farm to Pork at Slaughter
Source: Microorganisms. 2022 Jul 23;10(8):1485. doi: 10.3390/microorganisms10081485 (PMC9394271; doi:10.3390/microorganisms10081485)
Supplement: Supplementary file 1 [file microorganisms-10-01485-s001.zip › Table S1.pdf]

Table S1. Antibiotics applied at the respective farms during the study period, but not in study animals.

Antibiotic	Farm	Production mode
amoxicillin-trihydrate	E5	conventional
benzylpencillin-benzathin	E5	conventional
benzylpenicillin-procain	E5	conventional
cefquinome-sulfate	E5, J20	conventional, organic
chlortetacycline-hydrochlorid	E5, J20	conventional, organic
dihydrostreptomycin-sulfate	E5	conventional
enrofloxacin	E5	conventional
florfenicol	A6	conventional
lincomycinhydrochlorid-monohydrate	E5	conventional
spectinomycin-sulfate-tetrahydrate	E5	conventional
sulfadimidin + trimethoprim	J20	organic
sulfadoxin	E5	conventional
sulfathiazine + trimethoprim	J20	organic
tildipirosin	E5	conventional
tylosin-phosphate	A21	conventional
