# Supplementary material for: Tracking Antimicrobial Resistant E. coli from Pigs on Farm to Pork at Slaughter
Source: Microorganisms. 2022 Jul 23;10(8):1485. doi: 10.3390/microorganisms10081485 (PMC9394271; doi:10.3390/microorganisms10081485)
Supplement: Supplementary file 1 [file microorganisms-10-01485-s001.zip › Table S2.pdf]

Table S2. Overview of the primers used as well as their sequences and reference data.

No.	Primer FW/ RW	Target Gen	Sequence (Direction 5`-3`)	Sources
1	*adk*F<br>*adk*R	Adenylate kinase	ATTCTGCTTGGCGCTCCGGG<br>CCGTCAACTTTCGCGTATTT	http://mlst.warwick.ac.uk/mlst/
2	AMPC FW<br>AMPC RV	Chromosomal encoded *amp*C	GATCGTTCTGCCGCTGTG<br>GGGCAGCAAATGTGGAGCAA	Corvec et al. 2007
3	*bla*$_{CTX-M}$U FW<br>*bla*$_{CTX-M}$U RV	Conserved *bla*$_{CTX-M}$ region	ATGTGCAGYACCAGTAARGT<br>TGGGTRAARTARGTSACCAGA	Pagani et al. 2003
4	Com1<br>R789	16s-rRNA-Genes	CAGCAGCCGCGGTAATAC<br>ATCCTGTTTGMTMCCCVCRC	Schwieger et al. 1998<br>Dorn-In et al. 2015
5	ERIC 1 FW<br>ERIC 2 RV	ERIC	ATGTAAGCTCCTGGGGATTCAC<br>AAGTAAGTGACTGGGGTGAGCG	Versalovic et al. 1991<br>Chia et al. 2005
6	*fum*CF<br>*fum*CR	Fumarate hydratase	TCACAGGTCGCCAGCGCTTC<br>GTACGCAGCGAAAAAGATTC	http://mlst.warwick.ac.uk/mlst/
7	*gyr*BF<br>*gyr*BR	DNA gyrase	TCGGCGACACGGATGACGGC<br>ATCAGGCCTTCACGCGCATC	http://mlst.warwick.ac.uk/mlst/
8	*icd*F<br>*icd*R	Isocitrate/ isopropylmalate dehydrogenase	ATGGAAAGTAAAGTAGTTGTTCCGGCACA<br>GGACGCAGCAGGATCTGTT	http://mlst.warwick.ac.uk/mlst/
9	*mdh*F<br>*mdh*R	Malate dehydrogenase	ATGAAAGTCGCAGTCCTCGGCGCTGCTGGCGG<br>TTAACGAACTCCTGCCCCAGAGCGATATCTTTCTT	http://mlst.warwick.ac.uk/mlst/
10	*pur*AF<br>*pur*AR	Adenylosuccinate dehydrogenase	CGCGCTGATGAAAGAGATGA<br>CATACGGTAAGCCACGCAGA	http://mlst.warwick.ac.uk/mlst/
11	*qacE*aII FW<br>*qacE*aII RV	*qac*E / *qac*EΔ1	CGCATTTTATTTTCTTTCTCTGGTT<br>CCCGACCAGACTGCATAAGC	Jechalke et al. 2014
12	*qac*EΔ1 FW<br>*qac*EΔ1 RV	*qac*EΔ1	GGCTTTACTAAGCTTGCCCC<br>AGCCCCATACCTACAAAGCC	Bischoff et al. 2012
13	*rec*AF<br>*rec*AR	ATP/GTP binding site	CGCATTCGCTTTACCCTGACC<br>TCGTCGAAATCTACGGACCGGA	http://mlst.warwick.ac.uk/mlst/
14	*str*(A) FW<br>*str*(A) RV	*str*(A)	CCTGGTGATAACGGCAATTC<br>CCAATCGCAGATAGAAGGC	Lanz et al. 2003
15	*str*(B) FW<br>*str*(B) RV	*str*(B)	ATCGTCAAGGGATTGAAACC<br>GGATCGTAGAACATATTGGC	Lanz et al. 2003
16	*sul*(I) FW<br>*sul*(I) RV	*sul*(I)	TTCGGCATTCTGAATCTCAC<br>ATGATCTAACCCTCGGTCTC	Maynard et al. 2003
17	*sul*(II) FW<br>*sul*(II) RV	*sul*(II)	CGGCATCGTCAACATAACC<br>GTGTGCGGATGAAGTCAG	Maynard et al. 2003
18	*tet*(A) FW<br>*tet*(A) RV	*tet*(A)	GTGAAACCCAACATACCCC<br>GAAGGCAAGCAGGATGTAG	Maynard et al. 2003
19	*tet*(B) FW<br>*tet*(B) RV	*tet*(B)	TACGTGAATTTATTGCTTCGG<br>ATACAGCATCCAAAGCGCAC	Aminov et al. 2002
20	*tet*(M) FW<br>*tet*(M) RV	*tet*(M)	ACAGAAAGCTTATTATATAAC<br>TGGCGTGTCTATGATGTTCAC	Aminov et al. 2001
