# Supplementary material for: Tracking Antimicrobial Resistant E. coli from Pigs on Farm to Pork at Slaughter
Source: Microorganisms. 2022 Jul 23;10(8):1485. doi: 10.3390/microorganisms10081485 (PMC9394271; doi:10.3390/microorganisms10081485)
Supplement: Supplementary file 1 [file microorganisms-10-01485-s001.zip › Table S3.pdf]

Table S3. Target genes, fragment size and annealing temperature of the primers used for MLST-PCR

Number*	Primer FW/RV	Target gen	Fragment size (bp)	Annealing temperature (°C)
1	*adk*F<br>*adk*R	Adenylate kinase	583	54
6	*fum*CF<br>*fum*CR	Fumarate hydratase	806	54
7	*gyr*BF<br>*gyr*BR	DNA gyrase	911	60
8	*icd*F<br>*icd*R	Isocitrate/ isopropylmalate dehydrogenase	878	54
9	*mdh*F<br>*mdh*R	Malate dehydrogenase	932	60
10	*pur*AF<br>*pur*AR	Adenylosuccinate dehydrogenase	816	54
13	*rec*AF<br>*rec*AR	ATP/GTP binding site	780	58

* According to table S2
